# Trial to evaluate the immunogenicity and safety of a melanoma helper peptide vaccine plus incomplete Freund’s adjuvant, cyclophosphamide, and polyICLC (Mel63)

**DOI:** 10.1136/jitc-2020-000934

**Published:** 2021-01-21

**Authors:** Craig L Slingluff, Jr., Gina R Petroni, Kimberly A Chianese-Bullock, Nolan A Wages, Walter C Olson, Kelly T Smith, Kathleen Haden, Lynn T Dengel, Anna Dickinson, Caroline Reed, Elizabeth M Gaughan, William W Grosh, Varinder Kaur, Nikole Varhegyi, Mark Smolkin, Nadejda V Galeassi, Donna Deacon, Emily H Hall

**Affiliations:** 1 Department of Surgery, University of Virginia School of Medicine, Charlottesville, Virginia, USA; 2 University of Virginia Cancer Center, Charlottesville, Virginia, USA; 3 Public Health Sciences, University of Virginia School of Medicine, Charlottesville, Virginia, USA; 4 Office of Research Cores Administration, University of Virginia School of Medicine, Charlottesville, Virginia, USA; 5 University of Virginia School of Medicine, Charlottesville, Virginia, USA; 6 Department of Gynecology and Obstetrics, Emory University, Atlanta, GA, USA; 7 Medicine, University of Virginia School of Medicine, Charlottesville, Virginia, USA; 8 Cardiovascular Imaging Center, University of Virginia School of Medicine, Charlottesville, Virginia, USA

**Keywords:** adjuvants, immunologic, CD4-positive T-lymphocytes, antibody formation, melanoma, immunogenicity, vaccine

## Abstract

**Background:**

Peptide vaccines designed to stimulate melanoma-reactive CD4^+^ T cells can induce T cell and antibody (Ab) responses, associated with enhanced overall survival. We hypothesized that adding toll-like receptor 3 agonist polyICLC to an incomplete Freund’s adjuvant (IFA) would be safe and would support strong, durable CD4^+^ T cell and Ab responses. We also hypothesized that oral low-dose metronomic cyclophosphamide (mCy) would be safe, would reduce circulating regulatory T cells (T-regs) and would further enhance immunogenicity.

**Participants and methods:**

An adaptive design based on toxicity and durable CD4+ T cell immune response (dRsp) was used to assign participants with resected stage IIA-IV melanoma to one of four study regimens. The regimens included a vaccine comprising six melanoma peptides restricted by Class II MHC (6MHP) in an emulsion with IFA alone (Arm A), with IFA plus systemic mCy (Arm B), with IFA+ local polyICLC (Arm C), or with IFA+ polyICLC+ mCy (Arm D). Toxicities were recorded (CTCAE V.4.03). T cell responses were measured by interferon γ ELIspot assay ex vivo. Serum Ab responses to 6MHP were measured by ELISA. Circulating T-regs were assessed by flow cytometry.

**Results:**

Forty-eight eligible participants were enrolled and treated. Early data on safety and dRsp favored enrollment on arm D. Total enrollment on Arms A-D were 3, 7, 6, and 32, respectively. Treatment-related dose-limiting toxicities (DLTs) were observed in 1/7 (14%) participants on arm B and 2/32 (6%) on arm D. None exceeded the 25% DLT threshold for early closure to enrollment for any arm. Strong durable T cell responses to 6MHP were detected ex vivo in 0%, 29%, 67%, and 47% of participants on arms A-D, respectively. IgG Ab responses were greatest for arms C and D. Circulating T-regs frequencies were not altered by mCy.

**Conclusions:**

6MHP vaccines administered with IFA, polyICLC, and mCy were well tolerated. The dRsp rate for arm D of 47% (90% CI 32 to 63) exceeded the 18% (90% CI 11 to 26) rate previously observed with 6MHP in IFA alone. Vaccination with IFA+ polyICLC (arm C) also showed promise for enhancing T cell and Ab responses.

## Introduction

Resistance to checkpoint blockade immunotherapy is commonly attributed to a lack of pre-existing T cell responses to cancer antigens.^1^ Thus, there is compelling need for methods to induce antitumor immunity in such patients. Cancer vaccines targeting either mutated neo-antigens or shared tumor antigens may accomplish this; however, a critical limitation of cancer vaccine technology is lack of consensus on optimal vaccine adjuvants, which are required to induce functional immune responses. Studies to optimize adjuvants and strategies cannot be performed efficiently with neo-antigens, because the patient specificity limits the ability to study effects in a controlled and meaningful manner across a sufficient number of patients. Cancer vaccines inducing antigen-specific CD4^+^ T cell responses are emerging as promising cancer immunotherapies.^2–4^ We have studied a vaccine incorporating six intermediate-length peptides that induce CD4^+^ helper T cell (T_H_) responses (six helper peptides, 6MHP) and which has clinical activity in patients with advanced melanoma.^5–10^ The melanoma-associated class II MHC-restricted peptides in the 6MHP vaccine represent melanocytic differentiation proteins and cancer-testis antigens. In prior trials, we have found these peptides to be immunogenic in most patients when administered with incomplete Freund’s adjuvant (IFA).^9–11^ In those studies using IFA as the adjuvant-induced T cell responses that were often transient or of low magnitude. Antibody (IgG) responses to the peptides have also been detected and have almost always been strong and durable. The clinical relevance of the IgG response is unclear since the target antigens are intracellular, but we suspect that they may help to opsonize the peptides to enhance antigen presentation by dendritic cells in vivo. We have found that patient survival was significantly longer for patients who developed both T cell and antibody responses by week 7, compared with this with only T cell or antibody responses (or neither).^6^

The IFA used with this and other vaccines is Montanide ISA-51. Montanide ISA-51 consists of a mineral oil base similar to IFA; however, the Arlacel A emulsifying agent of older formulations of IFA has caused reactions in the past and has been replaced with a purified mannoside monooleate called ‘montanide’, which appears safer. Murine studies have raised concern about T cell sequestration and dysfunction at vaccine sites with use of IFA as a vaccine adjuvant with short peptides, but those concerns did not apply to a longer 20-mer peptide.^12^ The peptides in 6MHP range in length from 14-mers to 23-mers. Even for shorter peptides, in a recent clinical trial, we found that IFA can induce strong and durable CD8^+^ T cell responses, which may be enhanced by inclusion of a toll-like receptor (TLR) agonist.^13^ Others have also shown that addition of a TLR3 agonist (polyICLC, Hiltonol) or a TLR9 agonist (CpG) to IFA enhances T cell and antibody responses to long or short peptides in cancer patients.^14–16^ The role of a TLR agonist for augmenting T cell and antibody responses to a dedicated helper peptide vaccine has not been evaluated and was one goal of the present study.

Cyclophosphamide (CY) has also been studied as a systemic adjuvant for cancer vaccines. CY doses lower than those used for tumor lysis have been reported to augment immune responses in mice and humans^17–20^ through several potential mechanisms,^18 21–25^ including decreasing regulatory T cells^26 27^ and supporting dendritic cell maturation.^28^ In preclinical studies, immunopotentiation has been reported with CY administered 1–7 days prior to vaccination.^29–32^ Prior human trials of immunomodulatory properties of CY have tested doses from 75 to 1000 mg/m^2^, with variable results.^19 20 23 25 33 34^ For patients with melanoma, pretreatment with 300 mg/m^2^ of CY was associated with augmented delayed type hypersensitivity responses to an autologous melanoma cell vaccine in sequential non-randomized studies.^19 33^ Prior human experience suggested that CY increased immunogenicity when administered 3 days prior to a cell-based vaccine, but those studies were non-randomized and were limited by semiquantitative immunological endpoints.^19 20 33^ Other human experience failed to identify changes in regulatory T cells with CY treatment,^35^ and one study identified negative effects of CY doses of 200 mg/m^2^ or greater on cellular immune responses to a breast cancer cell vaccine, proposing that lower doses may support immunogenicity.^34^ The largest experience has been with a dose of 300 mg/m^2^ prior to vaccination. Thus, in a prior randomized prospective trial, we evaluated that dose, administered once, 5 days prior to the first vaccine, but found that it had no significant effect on circulating CD4^+^ or CD8^+^ T cell responses.^11^ However, a very different dosing scheme for CY has shown promise, where T cell responses to peptide vaccines in patients with ovarian cancer appeared higher in patients receiving a metronomic dosing of very low dose CY over a 10-week period in addition to vaccine, compared with patients who received vaccine alone^36^ Metronomic scheduling of various drugs has had differential and beneficial effects in multiple settings and has been justified in particular for CY.^37^ A goal of the present study was to evaluate whether T cell responses to the helper peptide vaccines would be increased by combination with this regimen of very low dose CY in a metronomic schedule. Primary objectives were to assess safety and immunogenicity of 6MHP vaccines plus Montanide ISA-51, with or without polyICLC and oral metronomic cyclophosphamide (mCy).

## Materials and methods

### Participant eligibility

Participants at least 18 years of age were eligible, if they had biopsy-proven Stage IIB-IV melanoma (by AJCC v7), at original diagnosis or at restaging after recurrence, rendered clinically free of disease by surgery, other therapy or spontaneous remission within 6 months prior to registration. Participants with stage IIA melanoma were also eligible if they were high-risk (class II) by DecisionDx Melanoma gene expression test^38^ (Castle Biosciences, Friendswood, Texas). Participants may have had cutaneous, uveal, mucosal primary melanoma, or an unknown primary melanoma. Also required were: ECOG performance status 0–1, ability and willingness to give informed consent, at least two intact regional node basins, normal organ function, and absence of major autoimmune disorders. Participants were excluded for pregnancy, other concurrent cancer therapy, uncontrolled diabetes or autoimmune disorders requiring therapy.

After completing enrollment of 47 participants in this adjuvant therapy study, the protocol was modified to allow enrollment of participants to arm D who had resectable metastases, to enable neoadjuvant vaccine therapy with a tumor biopsy prior to vaccination, then resection at day 22. Only one participant enrolled after the modification, and the data are all reported together.

### Vaccine components and treatment regimen

**Figure 1:**
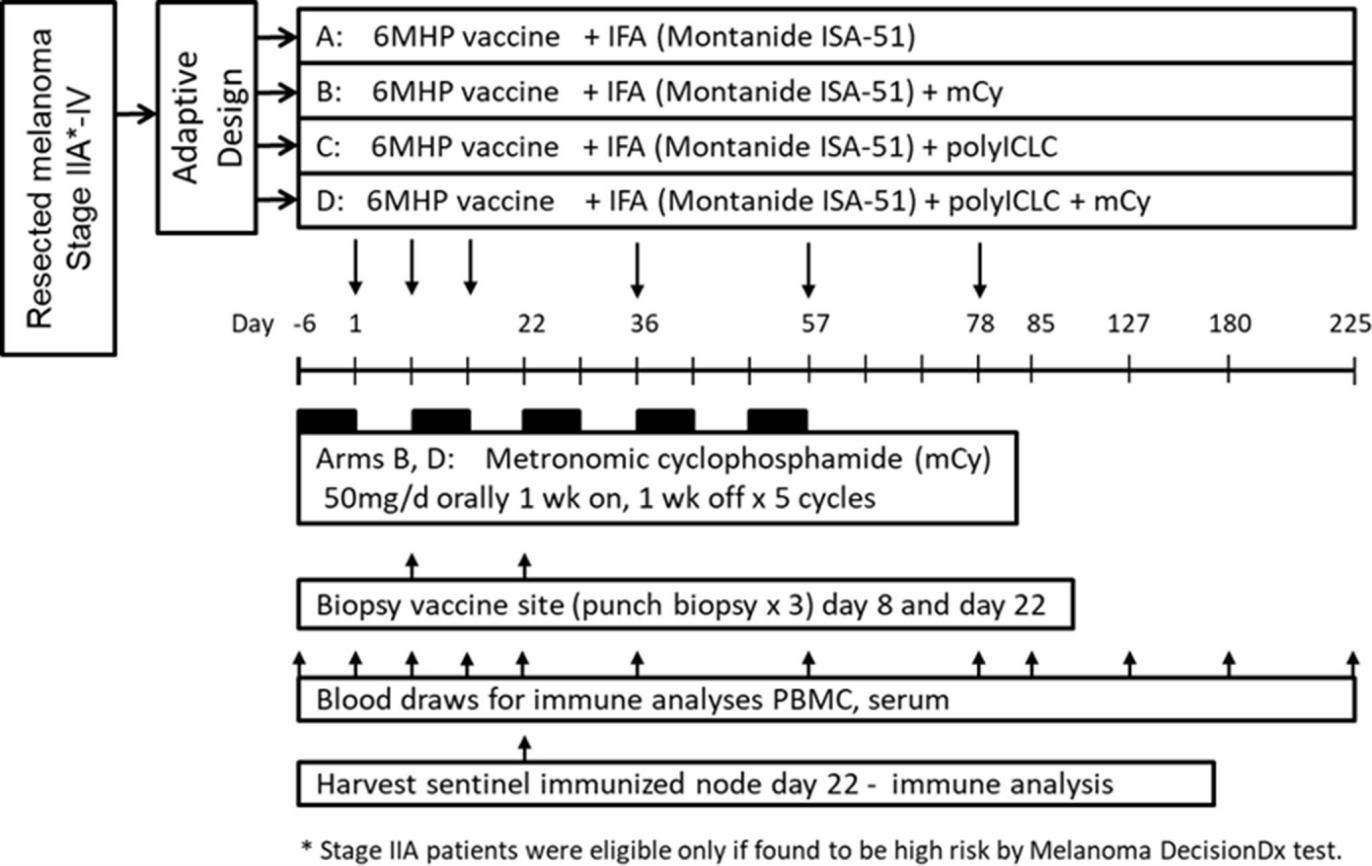
Study schema. IFA, incomplete Freund’s adjuvant; mCy, metronomic cyclophosphamide; PBMC, peripheral blood mononuclear cells.

The 6 Class II MHC-restricted melanoma peptides (6MHP) were synthesized and purified (>95%) in GMP conditions (Multiple Peptide Systems, now Polypeptide Group, San Diego, California, USA) and solubilized, sterile filtered and vialed as a mixture also under GMP conditions (Merck Biosciences AG Clinalfa; Läufelingen, Switzerland). The tetanus helper peptide (AQYIKANSKFIGITEL (p2_830–844_)) was synthesized and vialed under GMP conditions by same vendors. The single-use vials of peptide were tested for sterility, identity, purity, potency, general safety, pyrogenicity, and stability in accordance with Code of Federal Regulations guidelines. Methods for testing identity and stability of these peptide preparations have been reported.^39^ All participants were vaccinated with a mixture of 200 μg of each of 6MHP^40^ (online supplemental table 1) in one of two local adjuvant combinations (IFA alone, or IFA+polyICLC), with or without systemic oral low-dose mCy (figure 1). The IFA used was Montanide ISA-51VG adjuvant (Seppic, Inc, Fairfield, New Jersey). PolyICLC was provided by the Cancer Research Institute/Ludwig Institute for Cancer Research (New York), who purchased it from Oncovir (Washington, DC). When polyICLC was used, 0.5 mL (1 mg) was mixed with peptides before emulsification in IFA. For all vaccine preparations, peptides (with or without polyICLC) in a 1 mL aqueous solution were emulsified 1:1 with 1 mL of IFA, for a total emulsion volume of 2 mL. Vaccines were administered half-subcutaneously and half-intradermally in one skin location for the first three vaccines (days 1, 8, 15). The vaccine site was moved to a different extremity for the last three vaccines (days 36, 57, 78). Participants assigned to receive mCy were provided 35 oral 50 mg doses of Cy, which they began on day −6, and continued at 50 mg per day for 1 week. The CY was held for 1 week, then the 1 week on 1 week off cycle was continued through day 57 (figure 1). Blood was collected at multiple time points, and biopsies were performed of vaccine sites at weeks 1 and 3, and of a vaccine site-draining lymph node (sentinel immunized node, SIN) at week 3.^41^ Peripheral blood mononuclear cells and SIN cells were viably cryopreserved in 90% fetal bovine serum/10% DMSO, and serum was also frozen for subsequent immunologic assays in batch.

10.1136/jitc-2020-000934.supp1 Supplementary data

### Study design

The study was not designed to make definitive comparisons between arms. The primary objective of this study was to estimate the range of optimal treatment combinations and to expand accrual within the acceptable range to determine the best overall treatment strategy, thus treating as many participants as possible on the best treatment. An optimal treatment combination was defined as one with an acceptable toxicity profile as measured by dose-limiting toxicities (DLT) and a high rate of early and durable immune response (dRsp) as measured by CD4^+^ T cell response to 6MHP during the time period of vaccine administration. An adaptive design was used to guide accrual decisions, with toxicity assessments and the potential for a dRsp characterizing the primary decision measures.^42^ Although not designed to make comparisons between arms, the factorial nature of the design allowed for preliminary assessment of the effects of mCy (A+C vs B+D) and polyICLC (A+B vs C+D). Detailed considerations in this study design have been published.

### Participant enrollment

Participants were enrolled in two stages. The initial stage accrued eligible participants, in cohorts of two, to arms with increasing protocol-defined zones, and the second stage allocated eligible participants based on a continual reassessment method (CRM) for combinations of agents.^43^ The minimum follow-up period for escalation between zones was 3 weeks after the initial vaccine.

### First stage participant allocation

The escalation plan for the first stage was based on grouping treatment combinations into ‘zones’. Zone 1 was arm A (IFA alone); zone 2 included arms B and C (IFA +mCy, and IFA +polyICLC); zone 3 consisted of arm D (IFA +polyICLC + mCy). Participants could be enrolled and assigned to open arms within a zone, but escalation did not occur outside the zone until the minimum follow-up period was observed for the first participant enrolled to an arm. Initial allocation within a zone was based on random allocation (1:1) among possible arms. Escalation to a higher zone occurred only when all arms in the lower zone had been evaluated, and no DLT had been observed. Allocation of participants to subsequent arms within the new zone followed the same strategy. This allocation strategy was followed for accrual to increasing zones until a participant experienced a DLT or a stopping rule was triggered. Once a DLT was observed, the second stage using CRM modeling began.

### Second stage participant allocation

The second stage allocated eligible participants based on a CRM modeling approach that accounts for both toxicity and immune response in combinations of agents. Toxicity assessment was based on the occurrence of DLTs, and immune response assessment was based on achievement of dRsp. The estimated DLT probabilities at each arm were used to adaptively define an ‘acceptable’ set of safe arms, based on which arms had estimated DLT rates below the 25% DLT threshold with high confidence. Once the set of acceptable arms was determined after each new accrual, the recommended arm for the next accrual was chosen at random from the safe set, with each acceptable arm weighted by its estimated dRsp probability. This weighted randomization scheme was employed for the first one-third of the trial. In the latter portion of the trial, the recommended arm for the next accrual was the acceptable arm with the maximum estimated dRsp probability. Additional details regarding the modeling approach have been summarized in a prior report.^42^

### Dose-limiting toxicities

A DLT was defined as any unexpected adverse event (AE) that was possibly, probably or definitely related to treatment and (1) ≥grade 3, (2) ≥grade 1 selected ocular AEs, (c) ≥grade 2 allergic reactions. Expected AEs for mCy included grade 3 leukopenia, lymphopenia, and neutropenia, and ulceration at a vaccine site that did not exceed 2 cm in diameter. AEs that led to treatment discontinuation were also considered a DLT even if they did not meet the prespecified criteria for a DLT. The DLT tolerance level was chosen to be 25%.

### ELIspot assays and definition of immune response at each timepoint

ELIspot assays were performed on PBMC and SIN lymphocytes after cryopreservation but without in vitro stimulation prior to the assay as reported.^11^ These are referred to as direct (or ex vivo) interferon γ (IFNγ) ELIspot assays. Multiscreen HTS sterile 96-well polyvinylidene difluoride filter plates (Millipore) were prewet with 70% methanol, washed 3× with phosphate-buffered saline (PBS) and were coated with anti-IFN-γ mAb (Endogen) in PBS, and incubated overnight at 4°C. On the next day, plates were incubated 1 hour at room temperature (RT), washed × 3 with PBS, then blocked at least 1 hour at 37°C and 5% CO_2_ with complete media (RPMI +10% human AB serum +1%penicillin/streptomycin+1%L-glutamine). Effector cells were plated at 200,000 PBMC per well, and they were pulsed with synthetic peptide (10 μg/mL of each peptide), in quadruplicate. Plates were incubated at 37°C and 5% CO_2_ for 18–20 hours. Plates were then washed 6×. After extensive washing with 0.01% Tween, with 5 min incubation between washes, followed by rinse with triple deionized water. The plates were then incubated 2 hours at RT with a biotin-labeled anti-IFN-γ Ab (Endogen). Plates were washed 6× again, as above, and incubated 1 hour at RT with streptavidin conjugated to alkaline phosphatase (BD Pharmingen). After a final set of 6 washes with 0.01% Tween followed by triple deionized water, the plates were developed with NBT/5-bromo-4-chloro-3-indolyl phosphate substrate-Toluidine salt (Pierce), then rinsed with deionized water and dried. Plates were read using an automated plate reader (Bioreader; Biosys).

Negative controls included irrelevant peptide from HIV gag, and no peptide. Positive controls included each of the following: a mixture of viral peptides (CEF peptide pool^44^ at 2 μg/mL), phorbol myristate acetate-ionomycin and phytohaemagglutinin. Because of the adaptive design, early data on immune response were assessed together in one assay after samples through week 3 were available. Assays for later time points also included repeat analyses of a prevaccine sample as a control. The final analyses include those initial assays for the early weeks plus the final assays for the later time points. Evaluation of T-cell responses was based on the following definitions at each assay time point:

N_vax_=number of T-cells responding to vaccine peptide; N_neg_=number T-cells responding to maximum negative control; R_vax_=N_vax_/N_neg_. For evaluations of PBMC, a participant was considered to have a T-cell response to vaccination (binary yes/no) at each time point after baseline, by direct ELIspot assay only if all the following criteria were met: (1) N_vax_ exceeded N_neg_ by at least 20/100,000 CD4 (0.02%), (2) (N_vax_ – 1 SD) ≥ (N_neg_ +1 SD), and (3) R_vax_ after vaccination ≥5× R_vax_ prevaccine, as described.

The proportion of responding cells per 100,000 CD4 T cells was calculated based on the proportion of CD4^+^ T cells (CD3^+^CD4^+^ by flow cytometry) among PBMC or SIN. CD3^+^CD4^+^ T cells represented a median of 41% of PBMC samples (n=527), and 57% of SIN cells (n=45, data not shown).

Fold increases less than 1 were set to 1 to indicate no response and to prevent overinflating adjusted fold increases due to prevaccine ratios less than 1, or division by 0, while not affecting the determination of response. Negative control values of zero were set to 0.1 spots/100,000 CD4 T cells to avoid dividing by zero in calculating fold increases. The threshold for defining an immune response at any time point (Rsp) for this study was raised, compared with our prior analyzes^11^ by requiring a 5× increase over background rather than 2×, while still requiring all the other criteria listed above to be met. Continuous measures of immune response denoted as fold increase must satisfy conditions (1–3) and were defined as the amount of R_vax_.

Assay consistency is represented by interassay coefficients of variation (CVs) calculated for the response of two normal donors to the CEF peptide pool. For the high responder normal donor, mean number of spots was 208/100,000 cells plated, and CV was 34%. A low responder normal donor was included in 8 of the 51 assays, for which the mean was 39 and CV was 25%.

### Immune response endpoints

The trial was designed to determine the optimal treatment strategy among the four study combinations, defined as the one combination estimated to have an acceptable toxicity profile as measured by DLT and a high rate of early and dRsp as measured by CD4^+^ T cell response to 6MHP during the time period of vaccine administration.

A dRsp to 6MHP was defined as a CD4^+^ T cell Rsp to the 6MHP peptides in PBMC over two consecutive time points during vaccination (days 0–85). On review of data from a prior trial with 6MHP plus IFA,^11^ we identified potential dRsps in 18% (90% CI 11% to 26%) of participants, which provided a baseline to evaluate dRsp in this study. Another reported endpoint was the induction of a Rsp at two or more timepoints, not necessarily sequential (Any2Rsp).

For hypothesis-testing, participants who discontinued protocol therapy for allergic reactions or AEs, disease progression, or noncompliance, prior to collection of all blood samples were considered immune response failures if no response was observed in evaluable samples. Point estimates and 90% CIs were calculated for all summary parameters. A target of 30 participants on the recommended optimal arm was chosen based on having sufficient information to determine if the optimal arm shows an increase dRsp rate compared with the baseline rate observed in the 6MHP arms of the Mel44 trial of 18% (90% CI 11% to 26%). If at least 13/30 (43%; 90% CI 28% to 60%) participants on the optimal arm experienced a dRsp, the results would be considered promising since the lower limit of the CI exceeds the upper limit from the Mel44 estimated rate.

### Flow cytometry

When cryopreserved PBMC were thawed for ELIspot assays, about 200,000 cells were also assessed by flow cytometry for the proportions of CD4+CD3+cells among PBMC or SIN and for proportions of regulatory T cells (CD3^+^ CD4^+^ FoxP3^+^ CD25^Hi^ CD127 ^lo/neg^) among total CD3^+^ cells. The antibodies used for surface staining included: CD3 Horizon v450 (UCHT1), CD8 APC-H7 (SK1), CD4 PerCP-Cy5.5 (RPA-T4) and/or CD4 Fitc (RPA-T4) from BDBiosciences (San Jose, California); CD25PE (BC96), CD127 Fitc (RDR5) or CD127 PerCP-Cy5.5 (HIL-7R-M221) from eBioscience/Invitrogen (San Diego, California); and CD39 PE-Cy7 (A1, Biolegend; San Diego, California). Aqua live/dead marker (Molecular Probes/Invitrogen) was used at 1:600 to exclude dead cells. For detection of FoxP3, cells were fixed after surface staining, using the Ebioscience FoxP3 transcription fixative/permeabilization (Fix/Perm) kit (eBioscience Foxp3/Transcription Factor Staining Buffer Set: Invitrogen; ThermoFisher Scientific). Cells were fixed 30 min at 4°C and washed in Perm buffer. Cells were blocked in 2% normal rat serum (Jackson ImmunoResearch Laboratories) 15 min at RT before adding predetermined amount of FoxP3 APC (FOXP3-APC Ab (PCH101, eBioscience) and incubated 45 min at 4°C. Cells were washed in Perm buffer followed by washes and suspension in PBS (with 0.1% BSA and 0.1% sodium azide). Controls included rat IgG2a APC isotype control and fluorescent-minus-one controls for CD25 PE and CD127 PerCP-Cy5.5. Cells were acquired on a FACSCanto II flow cytometer (BD Biosciences) maintained in the Carter Immunology Center at the University of Virginia. Flow Jo software (BD Biosciences) was used to analyze data. Gating of live lymphocyte populations was based on forward and side scatter parameters and exclusion of Aqua^+^ cells. CD4^+^ cells were gated on CD3^+^ live lymphocytes. The frequency of FoxP3^+^ T cells in the total T cell (CD3^+^) population was calculated from the frequency of FoxP3^+^ cells in the CD4^+^ CD25^+^ and CD127^-^ lymphocyte population. A sample gating strategy is shown in online supplemental figure 1).

10.1136/jitc-2020-000934.supp2 Supplementary data

For analysis of circulating T-regs over time, repeated measures models^45^ with an unstructured covariance matrix and the baseline value as a covariate would not converge. Thus, a model with a heterogeneous autoregressive 1 covariance matrix was applied and F-tests based on contrasts were used to assess the specified comparisons. The Proc Mixed procedure in SAS version 9.4 was used to obtain model estimates and to calculate F-tests based on the specified contrasts.

### ELISA

Ab responses to peptides were determined by ELISA assay, as described.^46^ In brief, 96-well half-area cluster plates (Costar) were coated with 6MHP (8.3 ng/well per peptide) or with a control peptide derived from HIV (gag_293–312_; FRDYVDRFYKTLRAEQASQE^47^). Negative control serum was included from two healthy donors. Peptides were diluted in carbonate buffer (pH 9.4). After overnight incubation at 4 °C, wells were washed and then blocked with 5% nonfat dry milk/PBS/Tween to prevent non-specific binding. After 2 hours at RT, blocking buffer was removed, and participant serum added in a fourfold dilution series beginning at 1:100. After overnight incubation, plates were washed, and secondary Ab (Southern Biotech Goat anti-human IgG AP conjugate) was added. After washing, Attophos substrate (Promega, Fisher Scientific) was added and incubated at RT for 30 min in the dark. To stop the reaction, 3N NaOH was added, and the plate read on a Fluorescent plate reader (Molecular Devices SPECTRAmax Gemini EM, excitation 450 nm, emission 580 nm housed in the Biomolecular Analysis Facility). The FORECAST function in Microsoft Excel was used to calculate the Ab titer of participants' sera based on readings from the four dilutions, as described.^46^ The titer is defined as the reciprocal of the serum dilution that yields a fluorescent intensity ten times greater than the cut-off value. The cut-off value is defined as the average fluorescence obtained from the first four dilutions of serially diluted normal donor serum (negative control). Antibody titers<100 were not considered positive. For participants with pre-treatment titers≥100, an induced response required an increase of at least 4× over the pre-existing titer. Analysis of Ab over time was based on fitting repeated measures models^45^ with an unstructured covariance matrix and the baseline value as a covariate on logarithm transformed data and using F-tests based on contrasts to assess the specified comparisons.

## Results

### Clinical characteristics

The study opened to accrual May 12, 2015, and closed to accrual June 21, 2018. Total enrollment was 48 participants, all of whom were treated and were evaluable. These included 30 males (63%) and 18 females (37%). Most participants had ECOG performance status (PS) of 0 (94%) and stage III disease at registration (71%), and 62% had stage IIIB or higher disease. Additional details are provided in online supplemental table 2).

### Toxicities and AEs

The most common treatment-related adverse events (trAE) were injection site reaction (100%), skin induration at vaccine sites (100%), fatigue (60%), nausea (29%), myalgia (21%), headache (21%), chills (19%), influenza like symptoms (19%), arthralgia (19%), skin ulceration at vaccine sites (19%), fever (15%), cough (13%) and lymphopenia (10%). Details are provided in online supplemental table 3. Maximum grades for the systemic trAEs were primarily grade 1 and were transient. Vaccine site reactions, induration, and ulceration were grade 2 in 56, 58, and 0% of participants, respectively, and were grade 3 in 19, 1, and 2%, respectively. Injection site reactions with ulceration were grade 3 but were not considered DLTs if the ulceration was less than 2 cm in diameter and did not require surgical debridement or antibiotic therapy.

Three participants experienced treatment-related DLTs, one in arm B (VMM1244), and two in arm D (VMM1230; VMM1259). VMM1244 developed a septic joint near a site of recent trauma, leading to grade 4 sepsis, which was considered only possibly related to mCy but unrelated to the peptides or adjuvants. The participant was taken off study after two vaccines and was considered a failure for immune response analyses. VMM1230 experienced nausea, fever, arthralgia, myalgia, and dyspnea, all at grade 2, none of which individually met criteria for a DLT, but the symptoms in aggregate were judged to be dose limiting, and the participant was taken off study after 3 vaccines. VMM1259 developed grade 3 ulceration at a vaccine site that met criteria for a DLT and was taken off study after the sixth vaccine. Overall, these DLTs represented 1/7 participants in arm B (14%), and 2/32 in arm D (6.3%). Neither exceeded the 25% DLT threshold for early stopping.

All 48 participants received one or more vaccines. Forty-four (92%) completed all protocol treatment. Two stopped early for DLTs (one participant with a DLT competed all six vaccines prior to the DLT). Two participants experienced melanoma relapse during active treatment and discontinued early (see Consolidated Standards of Reporting Trials diagram).

### CD4 T cell response to 6MHP

**Figure 2:**
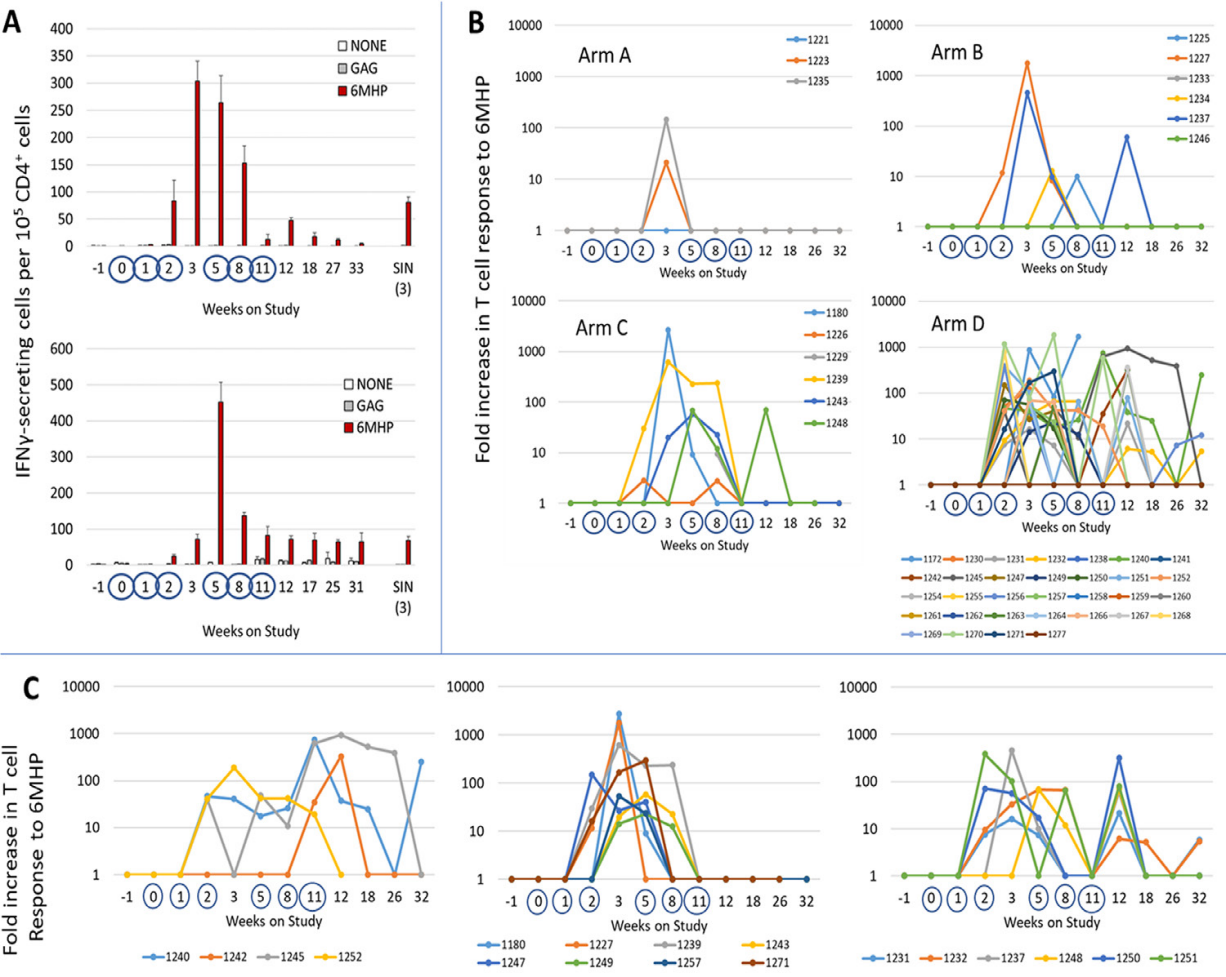
CD4+ T cell responses to 6 melanoma helper peptides (6MHP) by ex vivo interferon γ (IFNγ) ELIspot assay. (A) Examples of ELIspot data showing CD4^+^ T cell responses to 6MHP for patients in arms C (top panel, VMM1239), and D (bottom panel, VMM1232). Bars represent numbers of IFNγ-secreting cells per 10^5^ CD4^+^ T cells in peripheral blood (weeks −1 to 32) and in sentinel immunized node (week 3) after stimulation with the pool of 6MHP (maroon bars), no peptide (white bars, none), or HIV-gag negative control peptide (gray bars, gag). Dates when vaccines were administered are marked with blue circles. (B) T cell response data in PBMC for each patient, by study arm, and color coded by patient identifier (VMM number), shown as fold increase over background, corrected for any pre-existing response. Fold increases are shown only if all criteria for response are met (fivefold over background, 20 spots/10^5^ CD4^+^ T cells increase, no overlap of SD); thus, small increases over background are not shown, and the line is plotted along the X-axis. For arm B, among seven participants, one came off study too early to be evaluated for T cell response; so, data are shown for the other six participants only. (C) ELIspot data for participants with dRsp divided into those with response present week 11 (left panel), those without response weeks 11 or 12 (middle panel), and those without response week 11 that was restored week 12, with the 6th vaccine (right panel).

T cell responses were evaluated against the pool of 6MHP using direct ex vivo IFNγ ELIspot assays. The number (%) of participants evaluable for by ELIspot through weeks 3, 8, 11, 12, 26, and 32 were 47 (98%), 46 (96%), 44 (92%), 43 (90%), 39 (81%), and 37 (77%), respectively. Also, 43 (90%) were evaluable in the SIN. Examples of direct ELIspot responses to the 6MHP pool are shown in figure 2A. As evident in that figure, negative control values for each time point were usually very low. Among the 481 PBMC samples week 0 and later, median (and mean) values for the maximum negative controls were 0.5 (2.8) IFNγ-secreting cells per 1 00 000 CD4s, with 95% of samples having negative control values below 10. T cell responses for all patients, by study arm, are also shown as fold-increases over background in figure 2B. These images only show peaks above 0 when all criteria for a positive T cell response are met. Detailed data at each time point for all patients are provided in online supplemental table 4). Durable T cell responses (dRsp) to 6MHP in PBMC were identified in 0%, 29%, 67% and 47% of participants in arms A–D, respectively (table 1). T cell responses were most typically evident at weeks 2–8 but were often undetectable by week 11. To evaluate whether the vaccine at week 11 expanded putative memory T cell populations, we assessed those with dRsp (n=21) for whom samples were evaluable at both weeks 11 and 12 (n=18): of those 18, 4 (22%) had responses evident also at week 11 (figure 2C). However, 14 (78%) did not have responses at week 11: of these, 6 (43%) developed T cell responses at week 12, whereas 8 did not (figure 2C).

**Table 1:** T cell responses to 6 melanoma helper peptides (6MHP)

Arm	Durable T-cell response (dRsp) to 6MHP in PBMC*		Any 2 or more T cell responses (Any2Rsp) to 6MHP in PBMC†		# PBMC samples≥5×	Sentinel immunized node (SIN) responses	# PBMC or SIN samples≥5×	
	#/n (%)	(90% CI)	#/n (%)	(90% CI)	Min, max (median)	#/n (%)	(min, max)	Median
A	0/3 (0)	(0 to 63)	0/3 (0)	(0 to 63)	0, 1 (1)	0/3 (0)	0, 1	1
B	2/7 (29)	(5 to 66)	2/7 (29)	(5 to 66)	0, 3 (1)	2/6 (33)	0, 4	1
C	4/6 (67)	(27 to 94)	4/6 (67)	(27 to 94)	0, 4 (2.5)	4/5 (80)	0, 5	3.5
D	15/32 (47)	(32 to 63)	17/32 (53)‡	(37 to 68)	0, 8 (2)	9/29 (31)	0, 8	2

*Durable T cell response (dRsp) to 6MHP defined as T cell responses in PBMC at two or more sequential time points.

†Any2Resp is defined as at least two timepoints with a T cell response to 6MHP, not necessarily sequential.

‡VMM1256 (arm D) had responses at weeks 2, 26, 32, VMM1270 (arm D) had responses at weeks 2, 5, and 11.

Proportions with T cell responses to 6MHP at least 5× background at two time points (not necessarily consecutive, through day 225) were 0%, 29%, 67%, and 53%, respectively (table 1). T cell responses were also assessed in the SIN at week 3 and were detected in 0, 33%, 80%, and 31% of those evaluable, respectively (table 1). Each participant had PBMC evaluated at up to 12 times points (including weeks −1 and 0 prior to vaccine) plus one SIN sample. Thus, up to 11 samples were evaluable for vaccine-induced immune responses. The median numbers of these samples with immune responses, for arms A–D, respectively, were 1, 1, 3.5, and 2 (table 1).

### Changes in regulatory T cells

**Figure 3:**
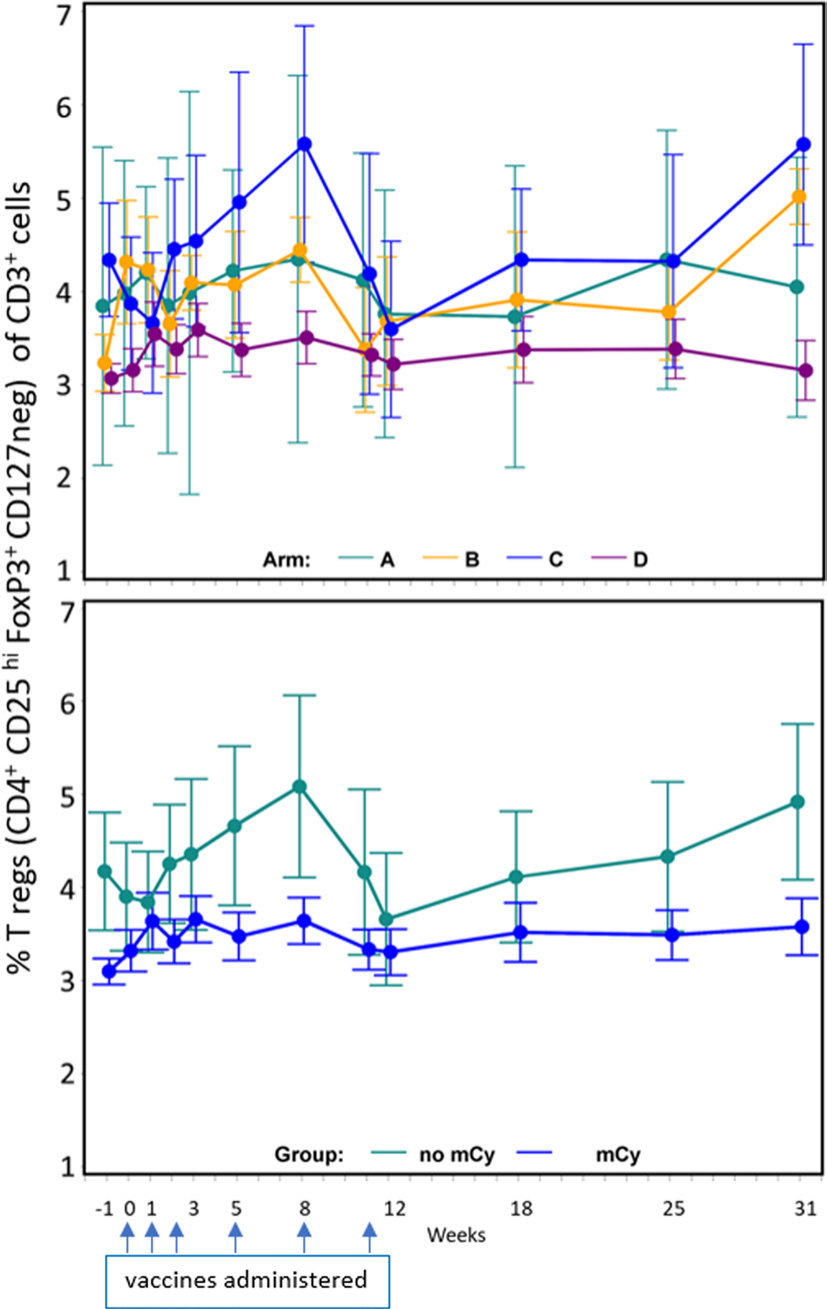
Impact of metronomic cyclophosphamide (mCy) on regulatory T cells over time. Regulatory T cells (defined as CD4^+^ CD25 ^hi^ FoxP3^+^ CD127 ^neg^) as a per cent of CD3^+^ cells in peripheral blood, means with SE bars. Top panel: data by arm. Bottom panel: data by use of mCy or not. Dates when vaccines were administered are marked with blue arrows.

Cy has been reported to decrease circulating T-regs, but it is unknown whether low-dose mCy will have that effect. We evaluated PBMC over time, identifying regulatory T cells as those expressing CD4, FoxP3, CD25(hi), and lacking CD127 expression, as a proportion of circulating T cells (CD3^+^). Participants randomized to receive mCy had significantly lower baseline T-reg values than those randomized to arms without mCy (p<0.001) (figure 3). No differences were detected in patterns of T-regs over time between those randomized to receive mCy and those not. The data for all evaluated samples (figure 3) for those treated with mCy showed no difference in T-regs between week −1 and week 0 (p=0.137); however, there was a significant increase in T-regs over time (weeks 0 to 8) compared with baseline (week −1) in participants treated with mCy (arms B and D) (p=0.030).

### Antibody responses to 6MHP

**Figure 4:**
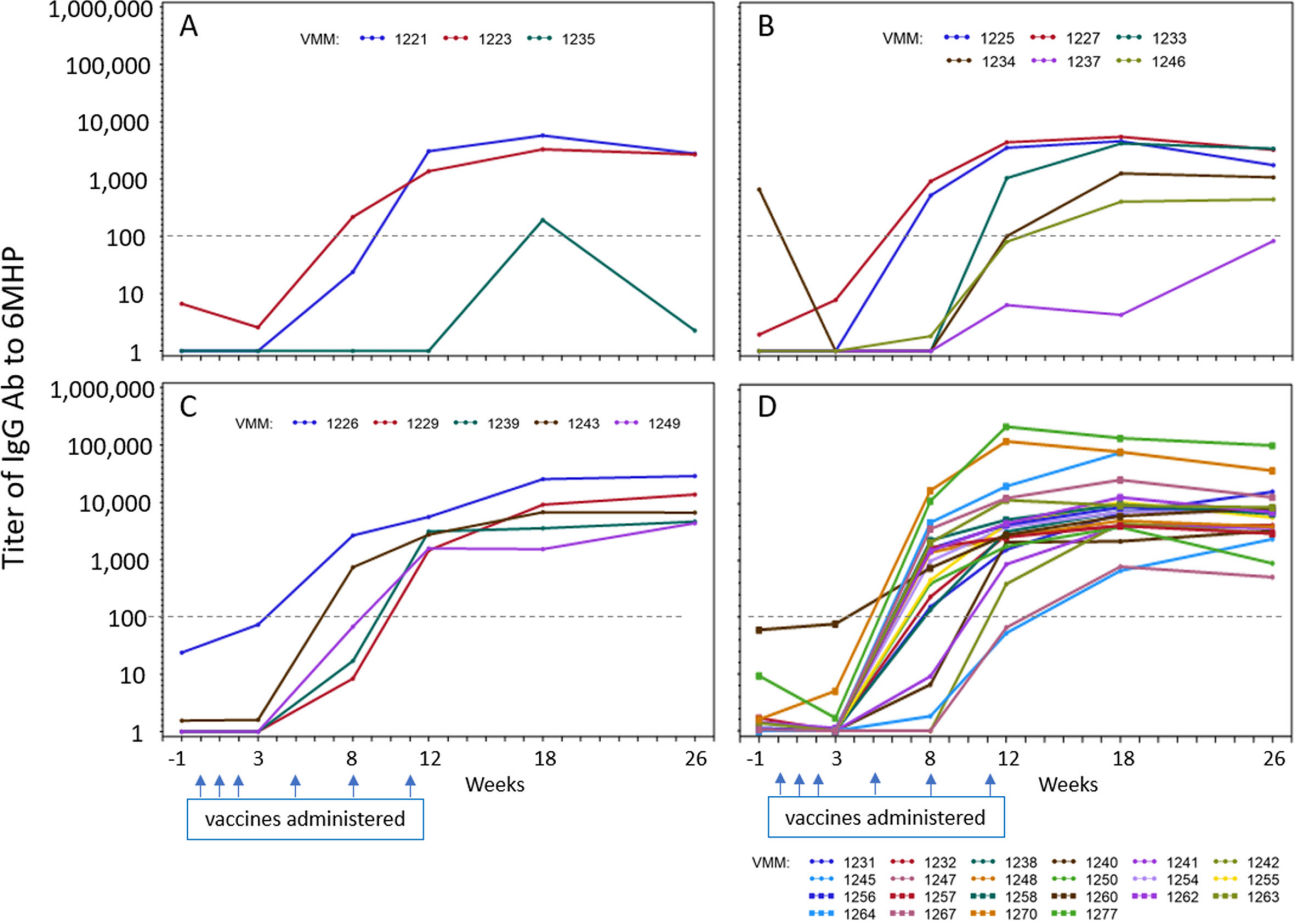
Antibody response to 6 melanoma helper peptides (6MHP). Serum IgG response to the pooled 6MHP over time from pre-treatment (week −1) through week 26. Titers for each patient are shown by a colored line, labeled by the VMM number. Panels A–D=arms A–D. The titer cut-off of 100 is shown as a dotted line. Dates when vaccines were administered are marked with blue arrows.

In prior work, with vaccines containing 6MHP plus IFA and GM-CSF, we identified strong IgG antibody responses to 6MHP that were significantly associated with T cell responses and with improved overall survival (OS).^6^ IgG responses to 6MHP were evaluated for most of the participants on this study (36 total: 3, 6, 5, and 22, in arms A-D, respectively). Only one (3%) had a pretreatment antibody response (titer >100). Ab responses were induced in 3, 4, 5, and 22 participants, representing 94% of evaluable subjects (figure 4). One in arm A was weak and transient. The others tended to increase to week 12 and to level off through week 26. There was a significant difference in baseline titer measures among the arms (p<0.001). Evaluating the impact of polyICLC, there was a significant difference in means over the five postbaseline time points, with the average titer in those who received polyICLC having a higher average difference than those without polyICLC (p<0.001), but no significant effect of mCy was detected (p=0.48). The estimated interaction effect of mCy and polyICLC was not significant at the 5% level.

### Clinical outcome

**Figure 5:**
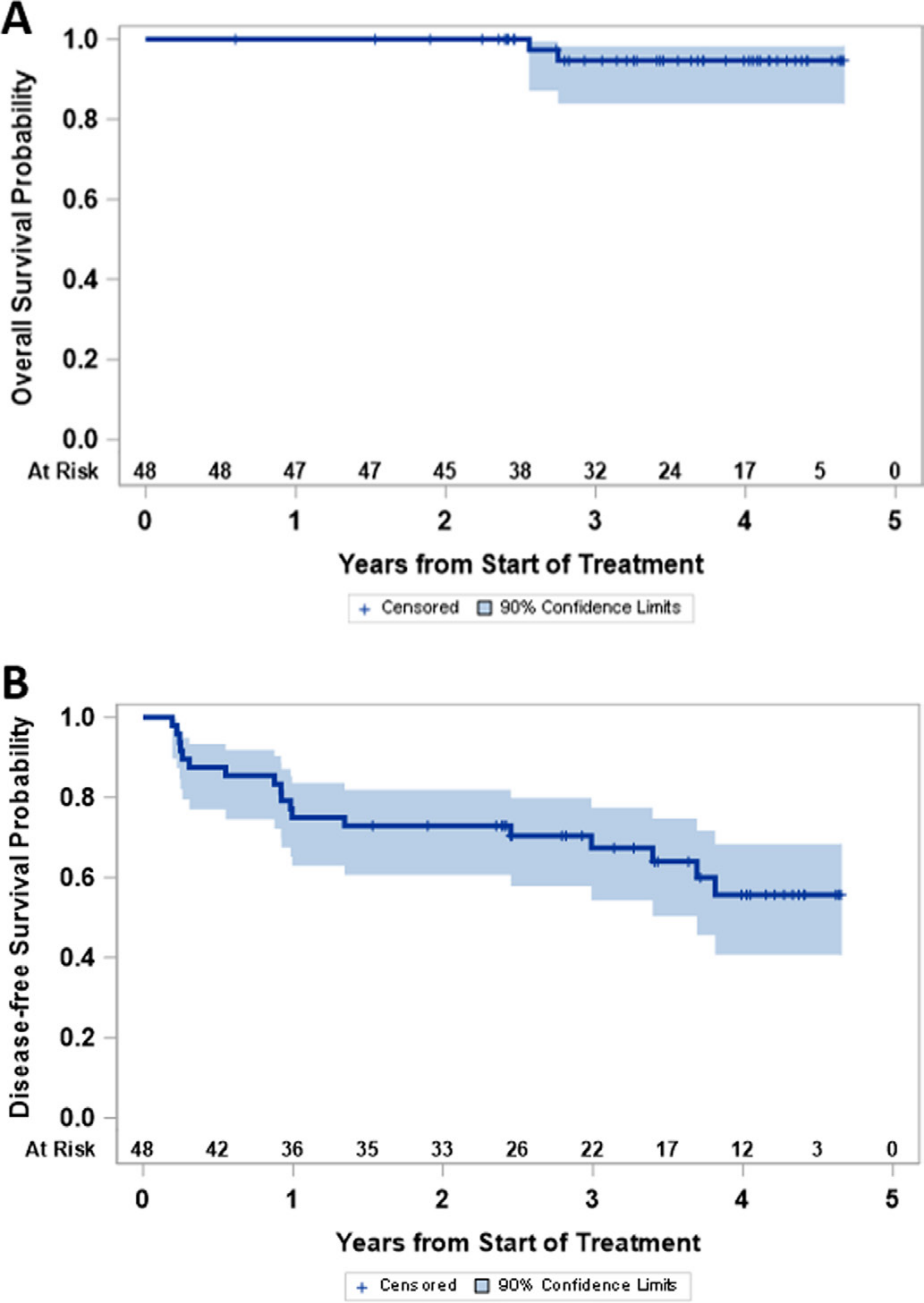
Clinical outcome: (A) disease-free survival and (B) overall survival are shown for the full study population.

The study was not powered for comparison of clinical outcomes among study groups, but OS and disease-free survival were high for the entire study population (figure 5). As of July 2020, 18 participants (38%) have experienced melanoma recurrence (2, 1, 1 and 14 on arms A, B, C and D, respectively), and 2 (4%) have died of advanced melanoma (both in arm D, 6.25%). Median follow-up for those still alive is 3.6 years. Four-year disease-free survival and OS are estimated at 56% (90% CI 41% to 68%) and 95% (90% CI 84% to 98%), respectively.

## Discussion

Immunotherapies targeting CD4^+^ T cell responses are showing promise, with clinical activity of cancer vaccines and of adoptive T cell therapies for melanoma and other cancers.^3 4 48^ Our prior work has demonstrated that vaccines using 6MHP emulsified in IFA have clinical activity^10^ and can induce CD4^+^ T cell responses in most participants, while also supporting induction of peptide-specific Ab responses.^6^ However, some T cell responses with IFA are transient, and not all participants develop strong responses. Thus, there is a need to enhance the immunogenicity of vaccines designed to induce melanoma-reactive helper T cells. In the present study, we have found that vaccination with these helper peptides in IFA +polyICLC, plus systemic mCy, induced durable T cell responses in 15 of 32 participants (47%; 90% CI 32 to 63), which was more than double the 18% dRsp rate with 6MHP+IFA alone in the prior Mel44 trial. Importantly, the 90% CI lower bound for dRsp of 34% exceeded 26%, the 90% CI upper bound for dRsp from Mel44, satisfying the prespecified criterion for evaluating the optimal recommended combination. Thus, the results for arm D support vaccination in an emulsion of IFA+ polyICLC as an effective local vaccination strategy with Class II MHC-restricted peptides, for inducing durable CD4^+^ T cell responses.

In addition to polyICLC, arm D included systemic mCy. However, it is not clear that the mCy contributed meaningfully to the immune responses. The overall dRsp rate for arm D was comparable (47%) to that of arm C, further supporting the value of IFA+ polyICLC, but challenging the contribution of mCy for use with this local adjuvant. Other measures of immunogenicity were similar for arms C and D, including the proportion with immune responses at any two (not necessarily consecutive) time points (67% vs 53%, respectively) and the number of samples with immune responses per participant (table 1). An advantage of the adaptive design used in this study was that it enabled enrollment to four study arms and selected the most promising arm for maximum enrollment, based on both safety bounds and early immune response data. However, the number of participants enrolled to arm C was only 6; thus, CIs for this arm are wide. Immune responses detected by ex vivo ELIspot assay were most commonly detected between weeks 2 and 8 and often decreased at later time points, even for those that met the criteria for a dRsp. Since the interval from the fifth vaccine on week eight to the blood drawn week 11 is 3 weeks, the lack of response at week 11 could be due in part to physiologic contraction of the T cell response, leaving a smaller pool of memory T cells. To evaluate whether the vaccine at week 11 expanded putative memory T cell populations, we assessed those with dRsp for whom samples were evaluable at both weeks 11 and 12: of those, 43% of the 14 without responses at week 11 developed responses at week 12. Thus, reductions in detectable T cell responses at week 11 or later may be explained in part by physiological contraction of the T cell response, which can be reactivated by repeat vaccination at week 11 in many cases. Since the blood draws at weeks 18, 26, and 32, were even further beyond the last vaccine, contraction of the responding T cell populations to memory may again explain diminished reactivity at the late dates.

No treatment arm presented with a DLT rate that exceeded the 25% DLT threshold that guided safety monitoring; thus, all arms were deemed safe. The DLTs varied, two consisting of toxicities that we have previously observed with vaccination,^11 49 50^ and one attributable primarily to an unrelated infection. No new patterns of toxicity were observed. In other experience, toxicities observed with higher doses of Cy have included hair loss and intravesical hemorrhage, but these were not observed in this study with low-dose mCy (online supplemental table 3).

The potential value of mCy for enhancing vaccine immunogenicity is supported by the selection of an arm that contained mCy (arm D) as the optimal arm based on the adaptive design, but further research is needed to determine the actual value of mCy for enhancing vaccine immunogenicity. In addition to T cell response data, arm D also had a high rate of early Ab responses (by week 8) (figure 4). Thus, these data leave open the possibility that mCy may enhance immune responses. One putative mechanism by which Cy may enhance immunogenicity is decreasing T-regs. Our data, however, do not reveal any significant reduction in circulating T-regs for arms B and D, and in fact identified a significant increase in T-regs over time in patients receiving mCy. The baseline T-reg levels were slightly lower in the participants on Arms B and D, which we consider to represent random variation among limited sample size. These findings do not rule out benefit by other mechanisms, which remain to be studied.

In prior work, we have identified strong IgG antibody responses to 6MHP and found that early Ab responses were associated with improved overall participant survival, especially when the participant also had a CD4^+^ T cell response to the peptides.^6^ Thus, we have investigated IgG Ab responses with the varied adjuvant regimens in this study. High rates of Ab induction were observed again, especially in arm D. The magnitude of the Ab responses was greater for Arms C and D than for arms A and B, supporting the value of adding polyICLC in the vaccine regimen, and consistent with prior work.^14^

The Kaplan-Meier curves for OS and disease-free survival (DFS) are presented and support very encouraging outcomes overall for participants on this trial, though the study was not powered for comparisons among treatment arms.

In addition to the primary and secondary endpoints of this study, which are the focus of the present report, this clinical trial included collection and preservation of biopsies from the vaccine sites and from the SIN from each participant. These will enable analysis of changes in the vaccine-site microenvironment that may help to understand the beneficial effects of IFA+ polyICLC and/or mCy in those tissues. Work is underway to study vaccine site biopsies and SIN from a range of clinical trials to put the findings in broad context. Also, for the one participant enrolled in a neoadjuvant setting, tumor tissue has been preserved prior to vaccination and after three vaccines. This will be studied as part of a larger effort to understand whether vaccine-induced T cells infiltrate melanoma metastases and remain functional in the tumor microenvironment.

The important findings from this trial thus far are that strong and durable helper T cell responses to their peptide antigens, detectable ex vivo, can be induced in about half of participants by vaccination with those peptides in an emulsion with IFA+ polyICLC, with or without mCy, and that all participants receiving these vaccines developed persistent Ab responses to 6MHP. This appears to be a more immunogenic strategy than vaccination in IFA alone, and supports prior work showing value of vaccination with IFA plus agonists for TLR3, TLR4, and TLR9.^13–15^ Also, this adjuvant formulation of IFA+ polyICLC is well tolerated, suggesting that the dose of 1 mg polyICLC in 1 mL IFA is reasonable for repeated use, even at the same injection site. In a prior trial of NY-ESO-1 long peptides, 4 of 11 participants treated with IFA+ 1.4 mg polyICLC per dose had marked grade 2 injection site reaction for which the investigators discontinued vaccines before the last dose: they suggested using a lower dose of polyICLC.^13^ In the present study, with IFA+ 1 mg polyICLC per dose, only one of 38 participants, on arms C+D combined, warranted early discontinuation due to injection site reaction. These data support the safety and immunogenicity with this dosing.

Questions have been raised about the value of vaccinating mice with emulsions with IFA, in particular with short peptides, restricted by Class I MHC.^11^ However, our own experience in humans supports the immunogenicity of IFA-containing vaccines, even with short peptides restricted by Class I MHC.^12^ The present study is novel in evaluating different vaccine adjuvant formulations for defined Class II MHC-restricted peptides, and it demonstrates the safety and immunogenicity of vaccination with these longer peptides for induction of helper T cells. These data add to findings from others for the value of TLR agonists in vaccine adjuvants. A hepatitis B virus vaccine (HEPLISAV-B) now incorporates a TLR9 agonist, and human clinical trials of cancer vaccines strongly support the value of adding a TLR9 agonist CpG-B to IFA.^15 16^ In our own experience with a different peptide vaccine, polyICLC combined with IFA appeared to support immunogenicity better than a TLR4 agonist combined with IFA.^13^ On the other hand, an adjuvant that incorporated agonists for both TLR4 and TLR9, but without IFA, appeared less promising in that T cell responses were only detected after in vitro stimulation.^51^ The present study, by inducing durable T cell responses detected ex vivo in 50% of participants supports use of polyICLC plus IFA as an adjuvant for helper peptide vaccines.

10.1136/jitc-2020-000934.supp3 Supplementary data

10.1136/jitc-2020-000934.supp4 Supplementary data
