# Supplementary material for: Trial to evaluate the immunogenicity and safety of a melanoma helper peptide vaccine plus incomplete Freund’s adjuvant, cyclophosphamide, and polyICLC (Mel63)
Source: J Immunother Cancer. 2021 Jan 21;9(1):e000934. doi: 10.1136/jitc-2020-000934 (PMC7825263; doi:10.1136/jitc-2020-000934)
Supplement: Supplementary data [file jitc-2020-000934supp001.pdf]

Supplemental Table 4. IFNγ-secreting cells per 100,000 CD4 T cells in peripheral blood and sentinel immunized node

		Week -1		Week 0		Week 1		Week 2		Week 3		Week 5		Week 8		Week 11		Week 12		Week 18		Week 26		Week 32		SIN: Week 3		dRsp in
VMM	Arm	C*	6MHP	C	6MHP	C	6MHP	C	6MHP	C	6MHP	C	6MHP	C	6MHP	C	6MHP	C	6MHP	C	6MHP	C	6MHP	C	6MHP	C	6MHP	PBMC?
1221	A	0	0	0	0.7	0.8	0.4	0.8	6.6	0.4	8.3	0.3	2.8	0.3	2.0	0.3	2.5	0.0	1.0	0.9	0.4	0.3	2.7	2.1	0.0	1.0	1.0	N
1223	A	0	0	0.3	0.0	1.3	1.3	0.3	11.9	**1.2**	**25.6**	0.3	4.8	0.0	0.5	4.6	2.5	1.4	0.0	6.4	4.4	4.7	4.1	3.8	7.6	0.8	11.4	N
1235	A	0	0	0.4	0.0	1.1	0.0	0.5	6.6	**0.3**	**44.3**	7.3	31.4	43.8	39.6	4.5	1.4	1.8	20.2	3.7	1.8	4.0	2.4	3.2	2.0	0.0	0.4	N
1225	B	0	0	0.9	0.0	0.3	0.0	0.0	0.3	0.9	1.9	0.3	2.9	**3.9**	**38.5**	0.3	0.0	0.7	0.0	0.4	0.0	0.0	0.0	3.1	0.0	0.0	0.0	N
1227	B	0	0	2.2	1.8	4.5	5.7	**3.0**	**35.1**	**0.0**	**229.5**	2.6	22.0	0.8	2.2	4.8	2.0	3.2	4.2	2.4	3.5	0.5	2.2	8.7	4.9	**0.5**	**40.1**	YES
1233	B	0	0	1.2	0.4	0.7	1.7	3.3	4.1	2.1	3.2	1.8	0.6	1.5	2.0	0.4	0.0	0.0	0.4	0.0	0.0	0.0	0.0	0.0	0.0	0.3	9.5	N
1234	B	0	0	0.7	0.4	0.0	0.9	0.0	0.0	0.9	10.4	**4.6**	**59.7**	1.8	3.4	1.4	0.3	0.5	1.6	1.7	0.8	1.5	0.5	2.8	0.9	0.3	2.1	N
1237	B	0	0	0	0.4	1.9	1.5	0.6	11.8	**0.6**	**288.7**	**2.7**	**27.0**	0.4	9.5	0.5	0.5	**0.4**	**22.8**	0.4	7.3	0.0	4.9	0.4	5.6	**8.1**	**113.5**	YES
1244	B	x	x	x	x	x	x	x	x	x	x	x	x	x	x	x	x	x	x	x	x	x	x	x	x	x	x	N
1246	B	0	0	0	0.0	0.3	0.0	0.3	0.3	0.0	0.4	0.0	1.2	0.8	0.8	0.0	1.1	0.0	1.2	0.0	0.3	1.0	0.6	0.0	0.0	0.0	0.0	N
1180	C	0	0	0	0.0	1.2	0.4	0.0	13.9	**0.0**	**229.4**	**2.9**	**107.2**	1.5	16.1	0.0	0.6	0.0	6.8	x	x	x	x	x	x	**0.3**	**44.4**	YES
1226	C	0	0	21.4	4.4	50.7	55.5	22.7	64.4	104.8	91.0	31.9	111.6	28.3	78.7	1.0	8.9	4.0	14.0	76.8	16.1	43.0	31.1	26.3	3.7	0.9	15.5	N
1229	C	0	0	1.2	0.4	2.5	2.2	11.0	8.7	9.8	36.2	x	x	**2.6**	**24.3**	5.4	3.3	4.4	8.6	6.3	5.5	3.2	3.2	3.3	6.6	x	x	N
1239	C	0	0	0.4	0.0	0.6	2.4	**2.9**	**83.3**	**0.5**	**303.9**	**1.1**	**261.2**	**0.6**	**152.6**	0.8	13.1	1.0	47.4	0.0	17.4	0.5	11.6	0.0	4.3	**0.9**	**81.0**	YES
1243	C	0	0	2.2	0.7	1.3	0.0	0.4	5.4	**1.6**	**32.0**	**0.4**	**113.3**	**0.7**	**79.9**	0.0	3.6	1.5	11.4	0.0	13.8	0.4	9.8	0.0	14.2	**0.0**	**26.2**	YES
1248	C	0	0	81.6	0.6	8.6	1.8	45.1	67.1	46.7	57.2	**0.6**	**39.0**	**2.1**	**25.1**	0.0	14.9	**0.4**	**29.2**	0.9	10.2	1.2	1.9	x	x	**2.6**	**125.8**	YES
1172	D	0	0	0.3	0.3	0.0	0.9	0.0	0.5	**0.3**	**251.7**	**0.4**	**38.0**	**0.0**	**155.8**	x	x	x	x	x	x	x	x	x	x	**0.0**	**143.6**	YES
1230	D	0	0	7.2	1.2	2.4	3.4	**5.0**	**257.0**	**3.7**	**454.2**	x	x	x	x	x	x	x	x	x	x	x	x	x	x	0.3	0.3	YES
1231	D	0	0	9.8	1.5	5.4	2.1	**14.7**	**111.8**	**3.2**	**51.7**	**2.0**	**42.3**	3.6	14.3	33.5	22.9	**2.6**	**73.5**	6.6	15.3	13.2	19.7	2.6	20.8	1.2	2.2	YES
1232	D	0	0	4.2	2.2	1.4	1.4	**2.6**	**24.3**	**2.2**	**70.8**	**6.8**	**451.6**	**2.1**	**137.0**	16.6	82.3	**11.9**	**72.6**	**13.4**	**69.2**	18.0	64.8	**12.0**	**64.6**	**1.3**	**67.5**	YES
1238	D	0	0	5.6	2.9	7.6	1.8	8.3	25.1	**1.9**	**133.3**	18.0	8.3	4.2	7.7	4.0	6.5	0.6	19.0	4.9	8.9	4.0	12.0	2.5	11.0	x	x	N
1240	D	0	0	1.3	2.1	0.8	0.0	**1.0**	**47.0**	**2.4**	**412.2**	**2.1**	**308.1**	**1.1**	**242.4**	**0.0**	**89.9**	**2.8**	**106.9**	**1.5**	**37.3**	0.0	12.0	**0.0**	**22.7**	0.3	15.0	YES
1241	D	0	0	0	0.4	0.0	0.0	0.4	4.2	0.5	17.8	0.0	1.2	0.0	8.2	0.0	2.9	0.0	0.7	0.4	2.0	0.0	5.5	0.0	1.8	0.0	3.7	N
1242	D	0	0	0	0.3	0.0	0.4	0.3	1.5	0.0	0.3	0.0	5.8	2.8	11.8	**1.3**	**42.2**	**0.0**	**27.4**	0.0	4.3	1.9	4.2	0.6	2.3	1.0	1.6	YES
1245	D	0	0	0.7	1.8	1.8	2.4	**0.7**	**26.5**	0.3	2.0	**0.6**	**59.2**	**1.2**	**25.8**	**0.0**	**63.7**	**0.0**	**93.8**	**0.0**	**53.4**	**0.0**	**37.0**	0.0	17.8	0.9	9.6	YES
1247	D	0	0	x	x	1.3	4.0	**0.8**	**122.3**	**1.4**	**37.2**	**1.2**	**46.6**	0.3	16.3	0.2	0.2	0.3	1.6	0.0	0.5	0.9	10.2	0.3	7.5	**0.5**	**30.0**	YES
1249	D	0	0	0	0.0	0.0	0.7	0.0	4.2	**0.9**	**56.7**	**2.3**	**256.2**	**2.7**	**164.4**	2.9	4.3	0.0	17.7	0.4	6.3	0.0	5.4	0.0	0.5	**0.3**	**39.1**	YES
1250	D	0	0	2.2	2.2	2.5	0.6	**1.8**	**122.5**	**2.7**	**152.1**	**0.6**	**88.6**	0.8	9.7	0.0	14.2	**0.0**	**24.4**	0.0	5.5	0.0	2.5	0.5	0.9	**0.3**	**45.5**	YES
1251	D	0	0	0.5	0.9	0.4	2.3	**0.0**	**99.0**	**0.5**	**103.5**	0.0	15.2	**0.4**	**30.1**	1.2	14.8	**0.5**	**44.6**	0.5	3.2	2.2	1.7	0.3	9.1	**0.3**	**44.6**	YES
1252	D	0	0	2.8	1.1	2.4	0.0	**1.0**	**43.3**	**0.4**	**84.2**	**5.9**	**247.4**	**3.6**	**150.0**	**1.1**	**21.1**	0.0	18.1	x	x	x	x	x	x	**0.4**	**49.6**	YES
1254	D	0	0	0.5	0.0	2.5	0.0	0.4	0.0	0.0	0.0	0.0	0.0	2.3	2.9	1.7	4.3	1.0	0.5	0.0	1.2	0.5	0.5	0.7	0.3	0.0	0.0	N
1255	D	0	0	0.8	0.4	0.0	0.0	0.0	1.6	0.3	0.9	0.0	2.4	0.0	2.2	0.0	1.7	0.2	7.2	0.6	4.9	0.4	4.1	0.3	0.0	0.5	0.2	N
1256	D	0	0	1.2	0.0	0.0	0.0	**0.0**	**45.6**	0.0	11.5	0.0	14.3	0.0	10.0	0.0	2.0	0.5	18.6	0.9	16.9	**2.1**	**30.6**	**1.2**	**27.9**	0.0	16.4	N
1257	D	0	0	1.1	0.7	1.3	4.4	1.6	4.3	**0.4**	**21.5**	**1.1**	**26.1**	0.4	9.2	1.6	17.6	0.4	3.4	0.0	3.1	0.4	2.0	0.0	1.8	**0.5**	**29.3**	YES
1258	D	0	0	0	0.6	0.0	0.0	2.0	2.6	0.0	1.6	0.6	7.7	0.6	0.6	0.6	1.1	0.0	0.5	0.0	1.0	0.0	2.8	0.0	1.4	3.0	9.8	N
1259	D	0	0	0	0.7	3.5	0.3	1.0	3.7	0.3	5.3	0.0	7.2	0.4	32.6	0.4	3.7	x	x	x	x	x	x	x	x	1.1	13.1	N
1260	D	0	0	0	1.8	2.5	0.0	0.4	1.9	0.5	0.0	6.6	0.5	6.8	1.1	0.5	1.1	0.0	0.0	0.0	0.4	0.8	0.8	1.5	0.3	0.9	7.5	N
1261	D	0	0	5.8	1.8	3.7	3.2	1.2	1.2	3.2	9.7	7.3	10.2	3.6	6.3	2.6	7.9	3.2	6.0	x	x	x	x	x	x	1.5	1.9	N
1262	D	0	0	0	0.0	0.0	0.4	0.0	0.0	0.4	19.4	0.4	15.5	0.0	4.1	0.4	13.2	0.9	19.4	2.6	11.1	2.0	1.6	1.7	1.7	0.3	13.5	N
1263	D	0	0	0.7	0.0	0.3	1.0	0.0	0.9	0.3	18.7	**0.7**	**34.8**	0.4	0.4	0.0	0.5	0.5	0.5	0.0	1.2	0.3	0.7	0.0	0.4	**0.0**	**33.6**	N
1264	D	0	0	0	0.6	0.0	0.7	0.6	0.3	**0.6**	**36.8**	0.0	8.3	0.0	10.7	0.0	12.1	0.0	16.7	0.4	2.4	0.4	0.8	0.0	0.5	0.3	12.4	N
1266	D	0	0	1.0	2.3	0.7	1.4	1.4	9.7	**1.7**	**114.3**	**0.7**	**43.4**	0.4	5.1	x	x	x	x	x	x	x	x	x	x	2.2	4.8	YES
1267	D	0	0	0.3	0.0	1.1	0.0	0.3	1.0	0.0	18.7	0.3	2.4	0.0	0.7	0.3	2.0	**0.0**	**34.9**	0.0	1.1	0.0	6.2	0.4	0.0	0.0	18.5	N
1268	D	0	0	0.3	0.0	0.0	0.4	**0.0**	**66.0**	0.0	2.3	0.0	10.8	0.0	0.4	0.0	10.8	0.0	11.8	x	x	x	x	x	x	0.3	14.9	N
1269	D	0	0	0.5	0.0	0.0	0.3	0.4	5.4	0.3	1.1	0.0	3.6	0.8	6.1	0.4	3.7	0.3	3.6	0.0	0.3	0.0	0.0	0.0	0.6	0.3	19.1	N
1270	D	0	0	0	0.0	0.4	1.6	**0.0**	**61.6**	0.2	16.9	**0.0**	**110.1**	0.0	7.9	**0.0**	**37.4**	0.3	8.7	1.1	16.1	0.2	4.4	0.0	3.7	0.3	4.9	N
1271	D	0	0	1.2	0.0	0.0	0.3	**1.5**	**23.6**	**0.3**	**49.2**	**0.0**	**21.8**	0.3	11.3	0.6	15.5	0.0	2.1	0.2	0.2	0.6	0.3	x	x	x	x	YES
1277	D	0	0	1.1	0.0	0.0	1.8	4.2	2.6	0.0	0.0	0.0	0.0	0.8	0.0	0.0	0.0	0.0	0.0	0.7	1.4	0.6	2.4	1.4	6.4	X	x	N

* C = negative control (maximum of 2-3 negative controls. All values are from PBMC except the last two columns. "x" marks samples not collected or not evaluable.
Samples meet criteria for T cell response when shown in **bold and underlined**.
