# Supplementary material for: Trial to evaluate the immunogenicity and safety of a melanoma helper peptide vaccine plus incomplete Freund’s adjuvant, cyclophosphamide, and polyICLC (Mel63)
Source: J Immunother Cancer. 2021 Jan 21;9(1):e000934. doi: 10.1136/jitc-2020-000934 (PMC7825263; doi:10.1136/jitc-2020-000934)
Supplement: Supplementary data [file jitc-2020-000934supp002.pdf]

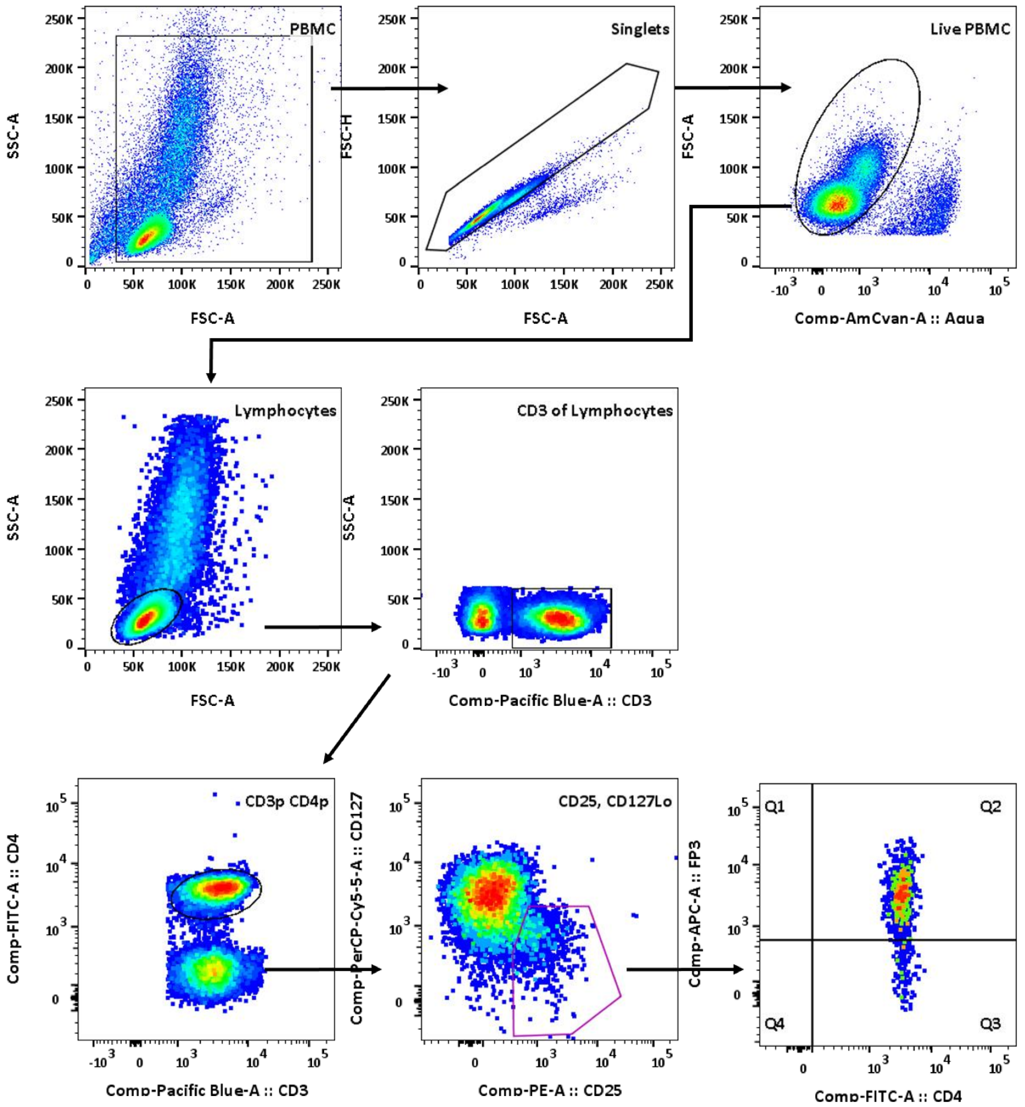

Supplemental Figure 1. Gating strategy for assessment of regulatory T cells. Gating of live lymphocyte populations in PBMC was based on forward and side scatter parameters and exclusion of dead (aqua+) cells. CD4+ cells were gated on CD3+ live lymphocytes. The frequency of FoxP3+(FP3+) T cells in the total T cell (CD3+) population was calculated from the frequency of FoxP3+ cells in the CD4+ CD25+ and CD127- lymphocyte population.
