# Supplementary figures and images for: Trial to evaluate the immunogenicity and safety of a melanoma helper peptide vaccine plus incomplete Freund’s adjuvant, cyclophosphamide, and polyICLC (Mel63)
Source: J Immunother Cancer. 2021 Jan 21;9(1):e000934. doi: 10.1136/jitc-2020-000934 (PMC7825263; doi:10.1136/jitc-2020-000934)

## CONSORT FLOW DIAGRAM

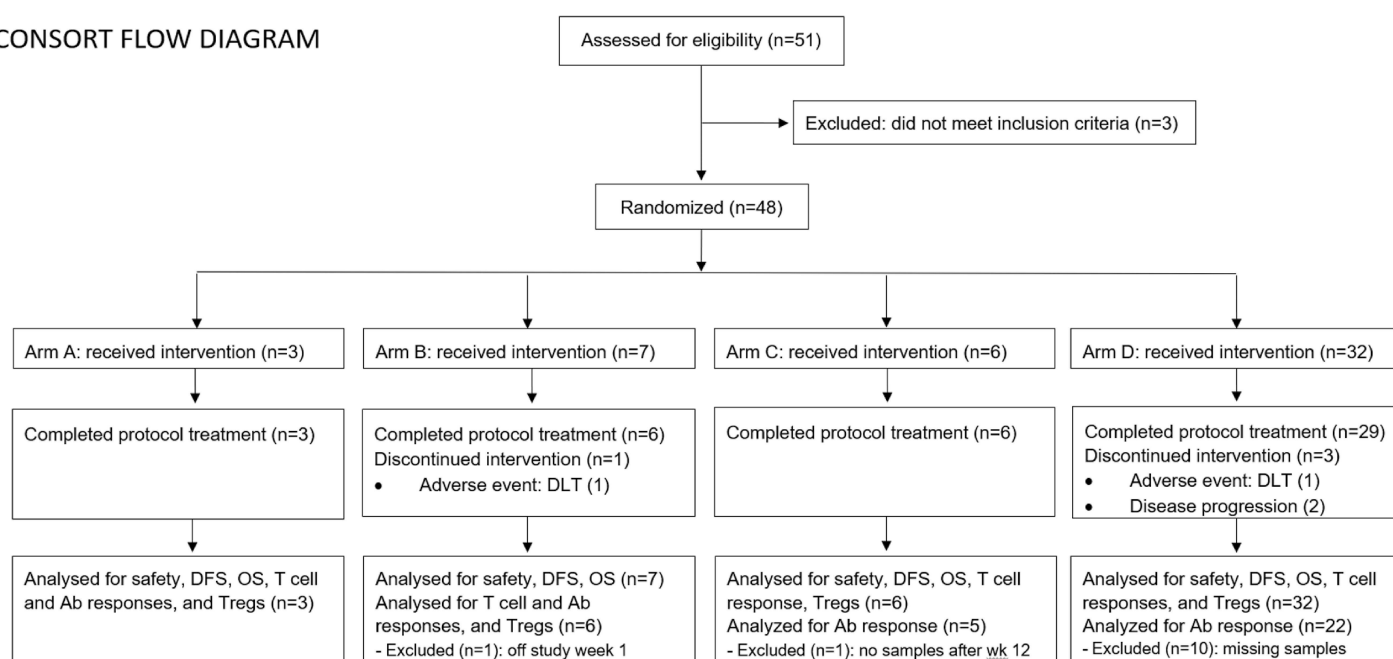

Supplement: Supplementary data [file jitc-2020-000934supp003.pdf]
