# Supplementary material for: Trial to evaluate the immunogenicity and safety of a melanoma helper peptide vaccine plus incomplete Freund’s adjuvant, cyclophosphamide, and polyICLC (Mel63)
Source: J Immunother Cancer. 2021 Jan 21;9(1):e000934. doi: 10.1136/jitc-2020-000934 (PMC7825263; doi:10.1136/jitc-2020-000934)
Supplement: Supplementary data [file jitc-2020-000934supp004.pdf]

Supplemental Table 1. 6 melanoma helper peptides

**Peptides used in the 6 Melanoma Helper Peptide (6MHP) vaccine**	
**Amino Acid Sequence**	**Epitope (source protein, residues)**
AQNILLSNAPLGPQFP	Tyrosinase $_{56\text{-}70}$ (alanine added at N-terminus)
FLLHHAFVDSIFEQWLQRHRP	Tyrosinase $_{386\text{-}406}$
RNGYRALMDKSLHVGTQCALTRR	Melan-A/MART-1$_{51\text{-}73}$
TSYVKVLHHMVKISG	MAGE-A3 $_{281\text{-}295}$
LLKYRAREPVTKAE	MAGE-A1,2,3,6 $_{121\text{-}134}$
WNRQLYPEWTEAQRLD	gp100 $_{44\text{-}59}$

Slingluff, Jr. CL, et al. J Immunother Cancer 2021; 9:e000934. doi: 10.1136/jitc-2020-000934

Supplemental Table 2. Participant Demographics

		Arm				Total
		A	B	C	D	
		6MHP + IFA	6MHP + IFA + mCy	6MHP + IFA + polyICLC	6MHP + IFA + polyICLC + mCy	
		N=3	N=7	N=6	N=32	N=48
Race	More than One Race	.	.	.	1	1 (2%)
	White	3	7	6	31	47 (98%)
Gender	Female	2	3	2	11	18 (38%)
	Male	1	4	4	21	30 (62%)
Ethnicity	Non-Hispanic	3	7	6	32	48 (100%)
Baseline ECOG Performance Status	Fully active (0)	3	7	6	29	45 (94%)
	Restricted (1)	.	.	.	3	3 (6%)
Primary site	Cutaneous	2	6	5	29	42 (88%)
	Mucosal	.	.	.	1	1 (2%)
	Unknown	1	1	1	2	5 (11%)
Stage at registration	IIA*	.	.	.	2	2 (4%)
	IIB	.	1	1	5	7 (15%)
	IIC	.	.	.	1	1 (2%)
	III, NOS	.	.	.	1	1 (2%)
	IIIA	1	2	1	3	7 (15%)
	IIIB	1	2	2	9	14 (29%)
	IIIC	.	2	2	8	12 (25%)
	IV	1	.	.	3	4 (8%)
Serum LDH	(missing)	.	.	1	4	5 (11%)
	LDH > ULN	.	2	1	4	7 (15%)
	LDH <= ULN	3	5	4	24	36 (75%)
Measurable disease at enrollment	No (adjuvant)	3	7	6	31	47 (98%)
	Yes (neoadjuvant)	.	.	.	1	1 (2%)

* Stage IIA participants were eligible only if their tumors were found to be high-risk (Class 2) by the Melanoma DecisionDx gene expression profiling test (Castle Biosciences).

Abbreviations: ECOG = Eastern Cooperative Oncology Group; NOS = not otherwise specified; LDH = lactate dehydrogenase; ULN = upper limit of normal (defined by performing laboratory)

Slingluff, Jr. CL, *et al. J Immunother Cancer* 2021; 9:e000934. doi: 10.1136/jitc-2020-000934

Supplemental Table 3. Treatment-related adverse events

IRB #17860 (MEL 63) Maximum Grade Toxicities (Related) OCT2018
# of subjects: 48

		Arms																				
		N=3 Arm A 6MHP + IFA				N=7 Arm B 6MHP + IFA + mCy				N=6 Arm C 6MHP + IFA + polyICLC				N=32 Arm D 6MHP + IFA + polyICLC + mCy				N=48 ----- Total				# (%) any grade
Category	AE	G1	G2	G3	G4	G1	G2	G3	G4	G1	G2	G3	G4	G1	G2	G3	G4	G1	G2	G3	G4	G1 – G4
Participant MAXIMUM grade	All		3				6		1		5	1		3	20	9		3	34	10	1	48 (100%)
BLOOD/LYMPHATIC	OTHER													1				1				1 (2%)
GASTROINTESTINAL	CONSTIPATION					1								1				2				2 (4%)
	DIARRHEA													1				1				1 (2%)
	MUCOSITIS ORAL					2								2				4				4 (8%)
	NAUSEA					2				4				7	1			13	1			14 (29%)
	VOMITING					1				1				1				3				3 (6%)
GENERAL AND ADMINISTRATION SITE	CHILLS									2				7				9				9 (19%)
	FATIGUE	2				2	1			4				18	2			26	3			29 (60%)
	FEVER					2								4	1			6	1			7 (15%)
	FLU LIKE SYMPTOMS					1				1				7				9				9 (19%)
	INJECTION SITE REACTION		3			2	5				5	1		10	14	8		12	27	9		48 (100%)
	PAIN													2				2				2 (4%)
IMMUNE SYSTEM	AUTOIMMUNE DISORDER													1				1				1 (2%)
INFECTIONS/INFESTATIONS	JOINT INFECTION							1												1		1 (2%)
	SEPSIS								1												1	1 (2%)
INJURY/POISONING/PROCEDURAL	BRUISING					1								1				2				2 (4%)
	WOUND COMPLICATION														1				1			1 (2%)
INVESTIGATIONS	LYMPHOCYTE COUNT DECREASED							1							3	1			3	2		5 (10%)
METABOLISM/NUTRITION	ANOREXIA					1								3				4				4 (8%)
MUSCULOSKELETAL/CONNECTIVE TISSUE	ARTHRALGIA	2				1	1							4	1			7	2			9 (19%)
	ARTHRITIS													1				1				1 (2%)
	MYALGIA	2								2				5	1			9	1			10 (21%)
	PAIN IN EXTREMITY					1												1				1 (2%)
NERVOUS SYSTEM	DIZZINESS													1				1				1 (2%)
	HEADACHE					2				3				5				10				10 (21%)
RESPIRATORY/THORACIC/MEDIASTINAL	ALLERGIC RHINITIS													1				1				1 (2%)
	COUGH	1												5				6				6 (13%)
	DYSPNEA						1								1				2			2 (4%)
	PNEUMONITIS													3				3				3 (6%)
	SORE THROAT													3				3				3 (6%)
SKIN/SUBCUTANEOUS TISSUE	HYPERHIDROSIS									1								1				1 (2%)
	OTHER	1								1								2				2 (4%)
	PRURITUS													1				1				1 (2%)
	RASH MACULO-PAPULAR					1												1				1 (2%)
	SKIN HYPOPIGMENTATION					1												1				1 (2%)
	SKIN INDURATION	3				6	1			2	4			9	23			20	28			48 (100%)
	SKIN ULCERATION									1				7		1		8		1		9 (19%)
VASCULAR	FLUSHING	1					1			1								2	1			3 (6%)
	HOT FLASHES													2				2				2 (4%)

Slingluff, Jr. CL, *et al. J Immunother Cancer* 2021; 9:e000934. doi: 10.1136/jitc-2020-000934
